# Semiautomated text analytics for qualitative data synthesis

**DOI:** 10.1002/jrsm.1361

**Published:** 2019-07-09

**Authors:** Emily Haynes, Ruth Garside, Judith Green, Michael P. Kelly, James Thomas, Cornelia Guell

**Affiliations:** ^1^ European Centre for Environment & Human Health University of Exeter Truro UK; ^2^ School of Population Health & Environmental Sciences King's College London London UK; ^3^ Primary Care Unit, Cambridge Institute of Public Health University of Cambridge Cambridge UK; ^4^ EPPI‐Centre, Department of Social Science University College London London UK

**Keywords:** data pooling, machine learning, qualitative data synthesis, secondary analysis, social practice, text analytics, text mining

## Abstract

Approaches to synthesizing qualitative data have, to date, largely focused on integrating the findings from published reports. However, developments in text mining software offer the potential for efficient analysis of large pooled primary qualitative datasets. This case study aimed to (a) provide a step‐by‐step guide to using one software application, Leximancer, and (b) interrogate opportunities and limitations of the software for qualitative data synthesis. We applied Leximancer v4.5 to a pool of five qualitative, UK‐based studies on transportation such as walking, cycling, and driving, and displayed the findings of the automated content analysis as intertopic distance maps. Leximancer enabled us to “zoom out” to familiarize ourselves with, and gain a broad perspective of, the pooled data. It indicated which studies clustered around dominant topics such as “people.” The software also enabled us to “zoom in” to narrow the perspective to specific subgroups and lines of enquiry. For example, “people” featured in men's and women's narratives but were talked about differently, with men mentioning “kids” and “old,” whereas women mentioned “things” and “stuff.” The approach provided us with a fresh lens for the initial inductive step in the analysis process and could guide further exploration. The limitations of using Leximancer were the substantial data preparation time involved and the contextual knowledge required from the researcher to turn lines of inquiry into meaningful insights. In summary, Leximancer is a useful tool for contributing to qualitative data synthesis, facilitating comprehensive and transparent data coding but can only inform, not replace, researcher‐led interpretive work.

**Highlights**

**What is already known?**

- There are increasing calls to make use of existing qualitative and quantitative data, increasing availability of large qualitative data and growth in demand for and approaches to data and evidence synthesis. Synthesis of large textual data is labor intensive and requires novel approaches.

**What is new?**

- We present the utility of text analytics as an independent method for contributing to qualitative data synthesis, facilitating more efficient, comprehensive, and transparent data familiarization and coding. The method enables analysis across various levels of supervision to modify in line with project objectives. It still requires researcher‐led interpretive analysis for meaningful results.

**Potential impact for RSM readers outside of the authors' field:**

- Text analytics software such as Leximancer can facilitate qualitative data synthesis of unusually large datasets in any field and invites further reflection and critique by social scientists.

## 1 INTRODUCTION

Evidence synthesis aims to draw transferable conclusions from often large and disparate datasets or research outputs to inform evidence‐based practice and policy decisions. In recent years, technological advances in automation have enhanced the efficiency of the review and analysis process. These advances have primarily focused on expediting the identification and synthesis of quantitative data.^1^, ^2^ However, qualitative evidence syntheses are increasingly conducted as stand‐alone or mixed‐method systematic reviews,^3^ and automation is less developed in these types of review. Current approaches to qualitative evidence synthesis such as thematic synthesis have been criticized for potentially decontextualising the findings of inherently context‐specific datasets.^4^, ^5^ Yet their value in contributing evidence about people's perceptions and experiences and the underlying mechanisms of quantitative findings are widely acknowledged.^6^, ^7^ While it is important to maintain the integrity of the primary research and to acknowledge its original context, qualitative *data* synthesis of raw primary data and qualitative *evidence* synthesis of primary research findings provide pragmatic and insightful approaches to produce evidence that is more transferable than that of individual context‐specific studies. Thus, there is a growing body of research and guidance that describe possible approaches to conducting and evaluating qualitative synthesis in meaningful ways.^8^, ^9^, ^10^ This paper focuses on one approach to synthesizing raw primary data pooled across studies and aims to provide a step‐by‐step guide to using one software application, Leximancer, for qualitative researchers less familiar with such tools.

Traditional approaches to analyzing or synthesizing the findings of large qualitative datasets are time and resource heavy. Expediting the process has been the subject of recent investigation, and potential approaches include the application of artificial intelligence and machine learning.^1^ Despite its conventional roots in quantitative data, the ability of machine learning and associated technologies to automatically and efficiently code large sets of data makes it potentially valuable for qualitative research, particularly given the recent increase in availability of large textual datasets within widely available public data repositories^11^ and other accessible platforms such as social media data.^12^, ^13^, ^14^ Inevitably, the benefits of applying machine learning to qualitative data are matched with limitations. Contrasting or conflicting language between machine learning experts and qualitative social scientists and the difficulty of capturing complex concepts using decontextualized features such as word occurrence are examples of the challenges of integrating automated techniques and qualitative data. Machine learning might, nonetheless, provide a framework for further exploration of relationships among the data and the opportunity to uncover networks or patterns that have not emerged from more traditional forms of researcher‐driven qualitative data analysis.

### 1.1 Automation or semiautomation of textual data

Several overlapping terms are used to describe software tools that might help in the analysis of textual data. *Text mining* is an umbrella term, which refers to the activity of retrieving information from unstructured text and then enabling users to view and interpret the results. There are numerous technologies used in text mining, which include natural language processing (NLP) and machine learning. The former tends to be used when the activity of programming computers to process text in semantically informed ways (eg, accounting for grammatical rules) is being considered. Machine learning refers to statistical approaches to text mining where the text is transformed into numeric form, and statistical interrelationships are analyzed.

Text mining has been applied to improve reviewing efficiency in systematic reviews and used to identify, categorize, and summarize data for rapid evidence synthesis.^2^ However, the application of text mining software to qualitative social science research has been limited to date.^15^, ^16^ Applications have largely been directed at the task of validation, or enhancing the credibility, of the findings of qualitative analysis in single studies.^17^, ^18^ The reluctance to apply text mining within primary qualitative research may stem from fixed perceptions of text mining as an inherently quantitative approach. However, text mining shares many commonalities with conventional qualitative content analysis, as an iterative, data‐driven approach that primarily focuses on “pattern recognition.”^16^, ^19^, ^20^ Thus, recognizing these commonalities might enhance support for applying machine learning as an appropriate and valuable tool to expedite the initial stages of “in vivo” coding and content analysis.

The approach to text mining used in this paper mostly utilizes statistical machine learning approaches. There are two common divisions of the machine learning: supervised and unsupervised. The two are distinguished by the level of input and a priori direction required from the researcher. The supervised approach requires researcher‐driven “rules” to inform an automated analysis. The machine learning algorithm is reliant on “training” (categorical ideas or theories given to the system) and then uses the learnings to code the full dataset. For example, the results of primary study analysis can be used to devise a classification scheme to synthesize further data. These approaches are most accurate when applied to large datasets and have been used in social and medical sciences to identify particular terms of interest within large volumes of social media data (for example, in datasets containing more than 600 000 tweets and posts).^21^, ^22^ A limitation of these supervised approaches is this need for prior codes or themes, which precludes the ability to uncover or reveal latent codes or themes that are not identified by the researcher.^23^

The unsupervised machine learning approach, on the other hand, does not require any rules, training sets, or key term dictionaries; structures and patterns are entirely driven from the input data and, in our case, transcripts. The process automatically extracts terms contained within the text or other data and develops a list of keywords; it performs the coding stage of the analysis without the need for any researcher input. Until recently, these analyses were based in simple algorithms to produce a list of words that are then used as labels to code the rest of the data. However, more recent iterations of these programs employ a more complex approach to identify not only lists of keywords but also interconnections with other words to identify “concepts” in context.^16^ They can quantify the interrelationships among terms, including how frequently they occur, how they interrelate with each other, and also in what contexts they interrelate. This unsupervised analysis of interrelating terms or “concepts” is known as “topic modelling analysis” and holds the potential for uncovering new and connected concepts within pooled datasets.

### 1.2 Leximancer

Leximancer is a text mining software application that was developed by researchers at the University of Queensland, Australia, to code automatically large qualitative datasets, and has since been validated and applied in various research dimensions.^24^ The software has been used in primary research to explore and develop definitions for terms such as “nutrients”^25^ and “disaster resilience”^26^ and to analyze opinion polls^27^ and transcripts from online discussion groups.^28^ It has been used to compare the conceptual similarity of perceptions between stakeholder groups^22^ and extended to explore interactional dynamics of real‐life conversations.^29^ The software has also been applied in systematic reviewing to select search terms^30^ and to track changes in abstract content within journals overtime.^31^, ^32^ However, to our knowledge, the utility of the software for qualitative data synthesis is yet to be explored.

Leximancer uses a defined set of terms to describe the various functions and analytic outputs, in particular “concepts” and “themes.” As these have different meanings in social science qualitative work, and to avoid confusion between the two “languages,” the Leximancer terms used in the context of this case study are explicitly defined in Table 1.

**Table 1:** Glossary of terms used by Leximancer

Term	Words in the text that have been examined for frequency of cooccurrence with other words and synonyms from the thesaurus and are weighted or scored according to evidence that a concept is present in a sentence.
Concept	Collections of words or “terms” that travel together within the text. They are parent terms that have been identified through semantic and relational word extraction that share similar meaning and/or space within the text.
Theme	Concept groups that are highly connected, parent concepts.
Importance	The hierarchy of “importance” indicates concept connectedness.

The text analytics tool performs an automatic unsupervised analysis of texts that are imported as individual files or folders. In analyzing the text, the system simultaneously conducts two forms of analysis: a semantic analysis that draws on the attributes of “entities,” words, or collections of words extracted by its own dictionary of terms and a relational analysis that draws on the frequency of occurrence.^24^ This builds a list of terms that are ranked according to their frequency of occurrence and interrelationships with each other. The system then draws upon the context of the terms to develop a thesaurus of interrelated terms, grouped by their semantic and relational connection, which become the “concepts,” and subsequently, interrelated “concepts” are merged to form the overarching parent concepts that are defined by Leximancer as “themes.” The initial result is a list of machine‐labelled key “themes,” constituting “concepts” and text excerpts from the data to support each concept. The text excerpts are grouped into chunks of two sentences and can be viewed in their original context to facilitate the interpretation of the data.

**Figure 1:**
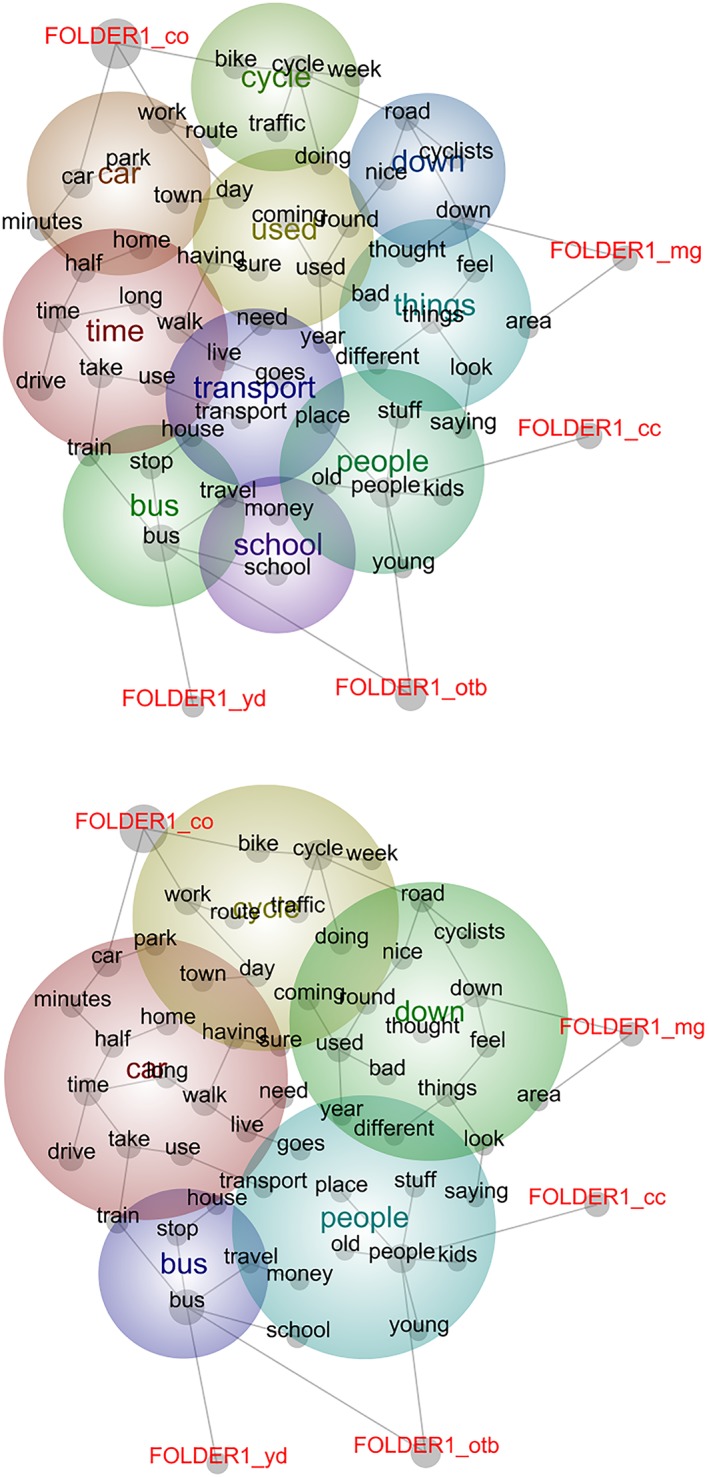
Presentation of findings tagged by primary study [Colour figure can be viewed at wileyonlinelibrary.com]

**Figure 2:**
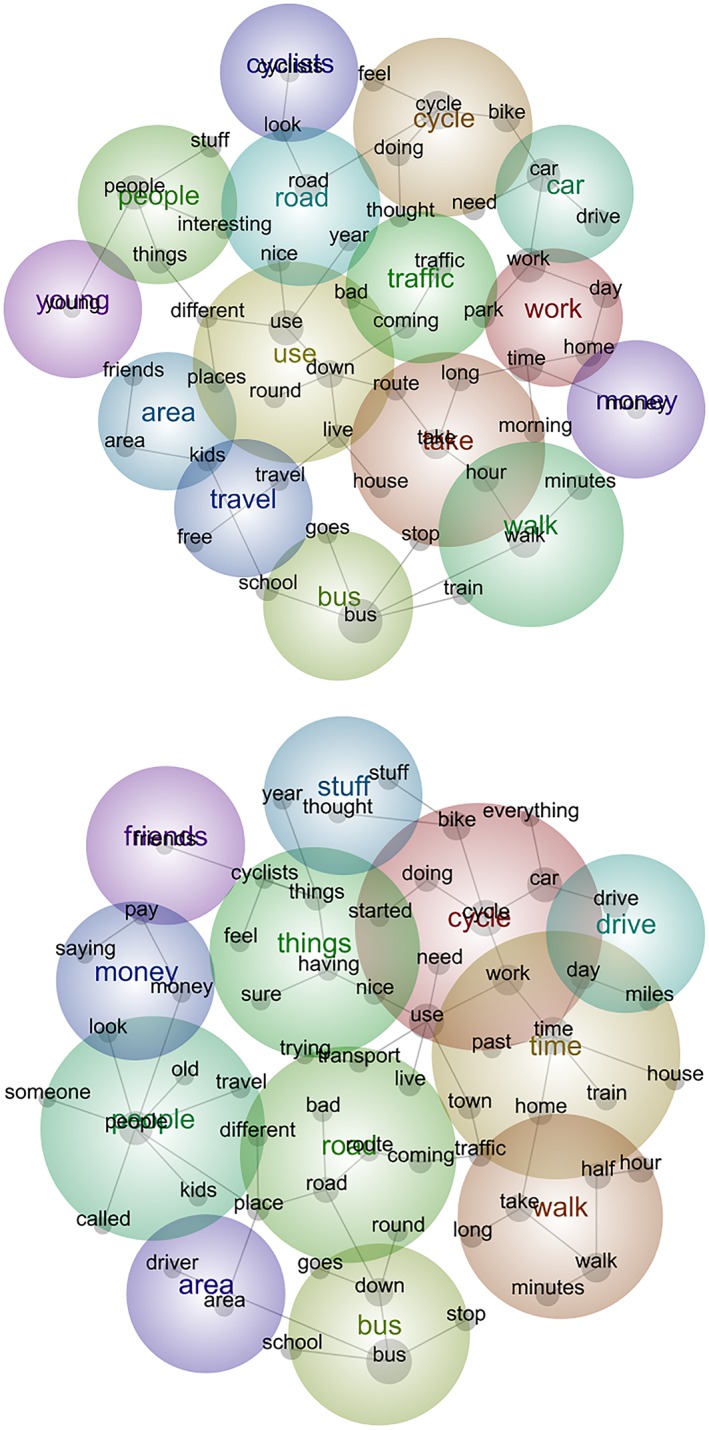
Presentation of findings classified by gender [Colour figure can be viewed at wileyonlinelibrary.com]

The outputs of Leximancer analyses can be presented in two ways. The first is a conceptual map (sometimes referred to as an intertopic distance map), which provides a bird's eye view of the semantic data. The key “themes” are illustrated as colored bubbles, and the colors are “heat mapped” to indicate relative “importance” or interrelatedness. Within the bubbles are collections of interlinked dots that represent the concepts that make up each theme. Tags can be allocated to specific data folders, files, or dialogue, and these tags displayed on the map in a similar way to the concepts. The proximity of the bubbles, concept dots, or tags to one another indicates conceptually similarity, with those clustered together most closely related. We present the results of our case study in this form (see Figures 1 and 2). The second visualization is a quantitative data summary that provides an overall bar chart of the data as frequency counts. The most frequent “themes” are displayed at the top of the chart, and the number of “hits” is indicated. Each theme links to a list of associated concepts, and five text extracts to support each concept are displayed; however, all text examples are also available to view if required. The bars are also heat mapped to correspond with colored bubbles of the conceptual map and to provide an integrative summary of the quantitative and semantic data (for example, see Data S1 and S2).

## 2 CASE STUDY: APPLYING LEXIMANCER TO SYNTHESIZE QUALITATIVE TRANSPORTATION STUDY DATA

### 2.1 Research design

In this case study, we describe how we applied an unsupervised machine learning approach to a pooled set of textual qualitative data from five primary research studies that explored practices and experiences of transportation, including everyday walking, cycling, driving and using public transport. Several of these relatively small‐scale studies had applied various social practice approaches to their investigation^33^, ^34^, ^35^ but cautioned that insights were clearly limited to their specific contexts and warranted further reflections on their transferability. Thus, the wider aim of applying a semiautomated text analysis approach to the pooled data was to uncover networks or patterns that have not emerged from the original and more traditional forms of qualitative analysis of the individual datasets. In doing so, we aimed to explore simultaneously Leximancer and its possibilities as an approach to qualitative data synthesis, which was the primary focus of this case study.

The dataset comprised 278 anonymized interview and focus group transcripts pooled from five UK‐based research studies. Study contexts ranged from commuting in Cambridge,^34^, ^36^, ^37^ cycling in London^38^ and free bus passes for young people in London,^39^, ^40^ to the impact of a new motorway in Glasgow^41^ and a graduated drivers license scheme in Northern Ireland.^42^ The studies included participants of various ages and gender and represented rural and urban locations across the United Kingdom.

We used Leximancer Desktop 4.5 to analyze our data and explore what the software can generate from a pooled qualitative dataset. Freely accessible training materials including tutorial guides, videos, and a detailed training manual were used to guide the analysis (https://info.leximancer.com/tutorial‐guides). Ethical approval for secondary analysis of the data was granted by the original ethics committees, where necessary, and overseen by the University of Exeter Ethics Committee as the lead institution.

### 2.2 Data analysis

The data analysis involved six key stages:

1. *Formatting transcripts*: Each transcript was edited to a standardized format in Microsoft Word to ensure compatibility with the software and to help Leximancer to distinguish between the interviewer and interviewee, as presented in the transcript template in Data S1. A unique identification number was developed using the basic contextual information available to us from the primary datasets and assigned to each anonymized transcript to enable mapping of gender, age range, location, study, and whether the transcript was derived from an interview or focus group.
2. *Classification of transcripts for analysis*: Each transcript was copied into relevant subfolders for analysis according to the participant's demographic information (gender and age range) and the study source.
3. *Automatic text processing and concept seed generation*: Tags were assigned at folder level for gender, age, and study to enable subgroup analysis (eg, women versus men and young people versus older people).
4. *Concept editing*: Only automatically defined concepts were used, and no tags or concepts were defined by the user. Identified concepts with limited relevance to the content of talk were removed, such as “probably,” “obviously,” and “yeah.” Plurals of concepts or those with similar meaning were merged (eg, car and cars, bus and buses, and cycle and cycling), and the thesaurus settings were set to program default.
5. *Concept coding*: The text was coded with “all discovered concepts” that were identified automatically and the folder tags that indicated the study, gender, and/or age of the participant related to the transcript. The decision was made to “kill” the name‐like concept “interviewer,” to suppress the processing of questions asked by the interviewer.
6. *Output*: The social network (Gaussian) map was chosen over the topic network (linear) map to emphasize the conceptual context in which the words appear and maximize the discovery of indirect relationships.

Table 2 details the step‐by‐step process taken in Leximancer and each command response provided during our analysis.

**Table 2:** Step‐by‐step process of analysis in Leximancer

Step	Process Options **(Our Command in Bold)**
1. Select documents	Select all transcripts or specific subfolders for subanalyses. **Folders relevant to each investigation (e.g. Age, Gender, Study)**
2. Text processing settings	Sentences per block: 1,**2 (normal)**,3,4,5,6,10,20,100 Prose test threshold: **0 (default)**,1,2,3,4,5 Duplicate text sensitivity: **Off**, Auto, 1,2,3,4,5,6,7,8 Identify name‐like concepts: **Yes**/No Break at paragraph: **On**/Off Auto‐paragraphing: On/**Off** Merge word variants: On/**Off** Tags: File, **Folder, Dialogue**
3. Concept seeds setting	Automatically identify concepts: **On**/Off Total number of concepts: **Automatic**, 10,20,30,40,50,60,70,80,90,100,110,120,130,140,150,160,170,180,190, 200,250,300,350,400,450,500,750,1000 Percentage of name‐like concepts: **Automatic**,10,20,30,40,50,60,70,80,90,100.
Generate concept seeds	
4. Edit concept seeds	Auto concepts or tags: Remove/merge any from list. Concepts removed **‘Yeah’ ‘laughs’ ‘obviously’ ‘probably’**. Concepts merged **‘car’ and ‘cars’; ‘bus’ and ‘buses’; ‘drive’ and ‘drives’; ‘cycle’ and ‘cycling’; ‘use’ and ‘uses’.** User defined concepts or tags: Remove/merge any from list: **None**
5. Thesaurus settings (concept learning)	Learn thesaurus from source documents: **Yes**/No Learn once: On/**Off** Concept generality: 1,2,3,4,5,6,7,8,9,10,11,**12 (default),**13,14,15,16,17,18,19,20,21. Learn from tags: On/**Off** Learning type: **Normal**/Supervised Sampling: **Automatic**,1,2,3,4,5,6,7,8,9,10 Sentiment lens: On/**Off** Number to discover: **Off**, 10,20,30,40,50,60,70,80,90,100,110,120,130,140,150,160,170,180,190,200,250,300, 350,400,450,500,750,1000 Themed discovery, concepts in **any**/all/each Only discover name‐like concepts: On/**Off**
Generate thesaurus	
6. Compound concepts	Choose any from list: **None**
7. Concept coding	Map: All names/all concepts/**All discovered names** (**specific folders only to represent sub‐analyses)/All discovered concepts/**All user names/All user concepts. Required concepts: From list as stated above—**None selected** Kill concepts: Choose from list of available concepts. ‘**Interviewer’**. Options: **All default settings.**
8. Project output settings	Map type: **social network**/topical network Default theme size percentage: 10,15,20,25,30, **33 (normal),** 35,40,45,50,55,60, 65 Map width: **Auto** Map height: **Auto**
Generate concept map	

## 3 RESULTS

As this paper aims to provide a guide to the opportunities and limitations of applying the software to qualitative analysis, we describe the findings of our case study through a process, rather than content, lens. We present our findings as two conceptual and interpretive insights of applying and reflecting on Leximancer, which we have called “zooming in” and “zooming out” to explore our pooled dataset.

### 3.1 Zooming out

In analyzing (large) qualitative datasets, it is important to be able to “zoom out” to gain a general overview of the textual data, familiarizing oneself with the data, and helping to map broad categories such as gender and age groups and broad shapes and patterns in the data. Leximancer delivers this overview as a visual, easy to read illustration. Figure 1 presents this “zoomed out” perspective of themes and constituting concepts derived from an analysis of all transcripts included in the pooled dataset. Here, the data have been organized and analyzed in subfolders according to each primary study. This facilitates data tagging to illustrate the clustering of concepts and indicate conceptual similarity and variation between the different datasets included in the synthesis (in this case between the studies). In this regard, tags can facilitate comparative analysis of the findings between any subgroup allowed by the demographic information available and providing that the data are arranged to distinguish between these subgroups.

In this example, Figure 1 shows clustering and thus greater conceptual connection between the transcripts from the Young Drivers (labelled as yd in Figure 1) and London bus pass (otb) studies around the theme of “bus” and “school.” The Cambridge commuters study data (co) were closely aligned to themes of “car” and “cycle,” while the Glasgow motorway (mg) and cycling in London (cc) studies were closely clustered around themes of “things” and “people” comprising concepts of “feeling,” “thoughts,” and “looks.”

Figure 1 also represents how the presentation of findings can be modified using a slider to adjust the grouping of concepts shown on the map. The slider presents fewer broader themes or a greater number of defined themes depending on the granularity required by the user. Zooming in and out in this way can help to uncover overlapping or dominant concepts retained by either resolution—in our case “people,” for example—or invite further exploration of the data to understand connections—in our case, for example, why “time” might be absorbed into “car” rather than its other connecting concept “bus.”

### 3.2 Zooming in

We explored “zooming in” as another important step of our synthesis. Leximancer also provides a platform to focus in on the data and follow lines of enquiry to analyze specific subgroups according to the available descriptors such as demographic information. We explored our data by “zooming in” on transportation as a gendered practice and divided the data by dialogue descriptors to provide two subgroups for analysis, men and women. Figure 2 illustrates how themes and concepts may vary between subgroups defined by gender.

In this example, the outputs indicate some conceptual similarity between the two subgroups, with 52% of identified themes common between the two groups; however, the maps and graphs (Figure 2 and Data S2) indicate that similar themes occur at varying frequencies and are made up of slightly different concepts when analyzed by gender. The program allowed us to identify similarities and differences between the subgroup findings. For example, the bar charts (Data S2) allowed us to identify that the theme “cycle” is of relatively similar importance (for explanation of “importance,” see Table 1) and frequency between the two subgroups and made up of similar concepts such as “doing,” “need,” and “bike.” In other words, these expressions (or synonyms or similar word stems) seem to “travel together” in the transcripts. The visual maps indicate that the theme of “time” is important and links those of “cycle,” “drive,” and “walk” in the men's narratives, while “time” is a constituting concept of work for women and themes of “road” and “traffic” are more closely clustered to the theme of “cycle” here. The exportable summary (Data S3a and S3b) indicates that the theme of “people” is made up of different concepts for men's and women's data. For men, “people” is composed of “travel,” “kids,” “old,” “someone,” and “called,” whereas for women, it is made up of “things,” “stuff,” and “interesting.”

Further interpretive analysis, however, then requires the qualitative researcher to return to the primary data. So, perhaps unsurprisingly, the two studies with young people clustered around similar themes in our “zooming out” analysis; but the two cycling studies did not relate conceptually according to Leximancer. Similarly, it may seem a counterintuitive finding that the concept of “kids” only strongly related to men and not women when “zooming in”.

Leximancer can also be a tool for this via specific functions for exploring concepts in context. For example, one useful function provides an exportable list of all text extracts that contributed to the development of a concept or theme, which can be used by the analyst to facilitate their interpretive work. Additionally, the software allows the analyst to investigate the cooccurrence of terms within the data. This function enables further in‐depth enquiry by allowing the analyst to “zoom in” on particular terms of interest. Finally, Leximancer can link any of these outputs to the original primary data in the transcripts, therefore simply serving the same data management function as other designated computer‐assisted qualitative data analysis software to aid sorting, exploring, and interrogating textual data. However, researchers might want to revert to these more commonly used software packages for these more familiar analysis steps.

## 4 DISCUSSION

In this paper, we provide a guide on how to use text analytics software, in this case Leximancer, to synthesize primary qualitative datasets. We provide a case study of using Leximancer to analyze a pooled dataset of UK transportation studies. Interrogating the process and utility of the software, we presented our findings as “zooming out” to gain an analytical overview of the data by broad categories of gender, age group, and study site available to us and “zooming in” to focus on specific subgroups of data and further explore, in this case, transportation as a gendered practice. In this discussion, we set out the opportunities and limitations of this software that we encountered in our case study.

### 4.1 Efficiency of analytical process versus labor‐intensive data preparation

The Leximancer software promises time efficiency, comprehensiveness, and relative ease of qualitative content analysis. It provides a user‐friendly platform, with functions that are easy to understand and apply to the data. Once settings are established, the analyses generate a concept map and data summaries almost instantly, compared with the labor‐intensive alternative of conducting such analysis by hand. A key advantage is the extensiveness of the analysis. Even with the support of computer‐aided qualitative data analysis software, comparing code or theme density is reliant on researchers' coding practices, which are inevitably shaped by a priori cognitive biases, theoretical frameworks, and a host of implicit heuristics, unknowable biases, and values.^43^ Unsupervised machine learning utilizes the entire dataset, with no preconceptions about how to code data extracts or what is relevant or not to a core category.

Despite the efficiency and extensiveness of the coding phase, it is important to consider the general efficiency of the process as a whole. One key consideration here is the initial challenge of obtaining and preparing the data from multiple studies. It was a time‐consuming process to navigate through various transcript coding systems, which were unique to individual studies, to develop a pooled table of demographic information. We then edited each transcript against a standard template (see Data S1) to ensure compatibility with the software and consistency across the pooled studies. In our case, to prepare our word files according to the template took an average of about 15 minutes per transcript and about 70 hours in total to prepare the transcripts and annotate the folders in Leximancer. The length of interviews, and therefore the size of the word files, varied greatly between and within studies. They were on average 69 KB, ranging from 21 to 215 KB, and between 2000 and 15000 words per file, with a total of 19.1 MB uploaded onto Leximancer. This process might, of course, vary greatly in other projects but is an important indication of the considerable time required to prepare the data.

### 4.2 Computer‐generated concepts and researcher‐led interpretations

Leximancer facilitates a highly inductive, data‐driven process, providing an analytical “fresh lens” and the potential for identifying novel linkages and groupings of specific terminology that might not be identified by manual coding. As an “unsupervised” method, the software relies on machine‐led pattern recognition in the concept generation and coding phase. By discounting researcher input in this phase of pattern recognition, the software does not allow for the grouping of more interpretative or theoretical ideas that could be related to one another. As we had very close knowledge of the used datasets, we deliberately opted for this approach to allow us to step back from previous analyses and research questions that shaped the original primary data collection and analysis. We aimed for this to generate new lines of potential enquiry, and the functionality of the software enables the researcher to follow such lines using subgroup analyses presented in both a broad or refined manner. For example, our case demonstrates the sensitivity of the software by illustrating how “themes” and “concepts” may change with the addition of a new dataset, in this case gendered subgroups. In turn, these findings may provoke further enquiry, for example, prompting questions such as in what context do women speak about traffic and cycling, to which Leximancer can facilitate further in‐depth investigation.

However, while this machine learning approach can uncover previously unanticipated patterns and clusters, researcher input and interpretative work is then necessary to make meaning from these. It is important to recognize that Leximancer only conducts the initial stage of the analysis and can only point to avenues for further interpretation. Regardless of the level of “machine learning” or artificial intelligence applied to data coding, the approach of the research in general should remain interpretative rather than aggregative, and therefore, understanding of the concepts still requires researcher‐driven interpretation.^44^, ^45^ Because of this, the fundamentals of interpretive qualitative analysis are preserved, and a Leximancer analysis raises the same interpretive considerations as purely researcher‐driven approaches to qualitative evidence synthesis. The software provides a helpful starting point to this interpretative work by providing a summary of text excerpts to support each concept that can be used to investigate what the findings of the initial Leximancer analysis actual mean in the context of the transcripts. Further interpretation of text excerpts is an essential phase to arrive at meaningful qualitative findings. We do not present findings from this further analytical work in this paper, but would like to emphasize that the software is a tool to facilitate the first steps of qualitative analysis, familiarization with and initial coding of large textual data, rather than a tool to replace the work of judgement, inference, and interpretation.

### 4.3 Levels of supervision and constraints of the original research

This analysis was intentionally focused on the unsupervised functions of Leximancer, given our aim of uncovering latent themes. However, the program also has the capacity to facilitate a range of more supervised machine learning approaches. The software allows the researcher to intermittently review the analysis, and at each stage of the process, we had to make “choices” (Table 2), which inevitably guided the findings. These functions allow the researcher to guide the findings by removing certain concepts from the analysis and enabled us to suppress the processing of interviewer questions and any concepts that we considered of limited relevance to the content of talk (eg, “obviously” and “probably”). Although these functions allowed for a more focused analysis, we acknowledge the limitations of these decisions and recognize that information about what the interviewer asked about or prompted for or the vocabulary used may provide valuable information for complementary analyses about interview content or conversational style.

If a more supervised approach is required, then analysts can define their own concepts or tags and direct the analysis to follow specific lines of enquiry. For example, we could have used the software to interrogate specific findings from the primary studies at greater scale across the pooled dataset. Alternatively, an initial unsupervised analysis may highlight conceptually similar terms through clustering, which can then be explored further for cooccurrence in the context of the text. For example, in the context of this case study, the findings could be used to explore the cooccurrence of the concepts “cycle” and “feels” to generate a pool of data for in‐depth enquiry around how people feel about cycling or cyclists. These semiautomatic investigations of identified terms may be particularly useful when working with very large volumes of data and support the value of the tool in wider contexts than that demonstrated by this case study.

The utility of Leximancer lies in this flexibility of the software to enable analyses of various levels of automaticity or supervision. We framed our analysis by subgrouping transcripts by the demographic information available to us and so to an extent have framed even this “unsupervised” analysis. This framing was guided by our own theoretical interests in the topic and previous research, in particular social practice approaches that understand transportation as a relational activity or behavior that tends to be performed or enacted with others, learned from others, and through the life course.^34^, ^35^ The relational character of Leximancer outputs seemed to promise a way of exploring such interrelations; and in addition, we anticipated that our demographic information on gender and age might further contribute to such a practice perspective.

The outputs of analysis are also inevitably constrained by the scope and content of the primary research studies, and the lacking contextual insight usually gained during data collection as a primary researcher. This is a feature of any method of data synthesis, given the findings are inherently bound to the specific contexts of the primary studies, and whatever question, sample or data generation limitations shaped their production. However, when pooled, as we have done here, we have the potential to compare across contexts and derive insights that speak to broader, varied contexts.

### 4.4 Reflections on terminology

In this case study, we have reported the functionality of the software and used Leximancer's explicitly defined terminology to do so. However, we previously highlighted that this language does not map neatly onto that of conventional qualitative research, in particular the use of the terms “themes” and “concepts.” This could cause confusion when interpreting the findings in the context of the transcripts and where both “languages” are used concurrently to conceptualize the findings. We have therefore attempted to describe and clarify how these Leximancer terms relate to common terminology of qualitative (thematic) analysis in the following way.

Leximancer's use of “term” refers to words within the text that have been examined for frequency of cooccurrence with other words and synonyms. These are weighted or scored according to evidence that a concept is present in a sentence, and therefore, “term,” as a basic unit of meaning, might map onto the use of an in vivo code in qualitative data analysis. A collection of these “terms” that travel together within the text are defined as “concepts” in Leximancer. These collections have been identified through semantic and relational word extraction that share similar meaning and/or space within the text. Therefore, Leximancer's “concepts” may be considered to be descriptive families of codes, or subcategories, in qualitative data analysis. In Leximancer's final stage of classification, emergent concept groups that are highly connected are defined as “themes.” These defining or conceptual labels for families of codes would be more commonly referred to as categories in traditional qualitative analysis as they lack the interpretive stage and theoretical framing of analysis.

Finally, the term “important” is used in Leximancer language, and the hierarchy of “importance” is defined as concept connectedness. In traditional qualitative data analysis, insights and findings are perhaps more likely described as interpretive or meaningful, for theoretical understanding of the data and identifying what is particularly pertinent or revealing in relation to the research question.

Indeed, these variations in language pose a threat to clarity in reporting the findings of qualitative data synthesis that use these text mining software applications. Future research using such programs should explicitly acknowledge these identified language differences when presenting their findings.

### 4.5 Future research and epistemological inquiry

Our exploration of a semiautomated text analysis software such as Leximancer suggests utility beyond our case of pooling a set of qualitative studies. Advancing communication platforms and growing qualitative data repositories give rise to large volumes of textual data becoming increasingly available to social scientists. The software could be particularly useful for exploring other data types such as social media or online blogs that produced large amounts of qualitative data.^14^

However, the application of such software should invite further critical exploration and reflections. For example, the software lends itself to explore the data more explicitly for conversational style and narrative analysis. The focus on terms and their cooccurrence might point more to deliberate or implicit narrative preferences and conventions than people's experiences. We also met our own limitations in understanding the extent of machine learning the software performed for us; for example, repeated running of queries results in different outputs as the software “learns” from the data when we used the unsupervised functions of the software. To get the same original outputs despite what Leximancer calls a stochastic process of generating maps (https://info.leximancer.com/tutorial‐guides), we learnt that a query needs to run “from scratch.” There seems to be a need for better integration of skills from social science and computer science to understand such “black boxes” of machine learning and algorithms for data and evidence synthesis.^13^ There are some intriguing parallels between the way that the software learns from the data and the way that both phenomenology and neuroscience describe the plasticity of human perception—the way that humans learn from the data and information they are exposed to.^46^ Alfred Schutz distinguished between ideal types as higher order organizing concepts and lower level more plastic typifications that are used to make sense of everyday life.^47^, ^48^ Typifications change and evolve as new information becomes available. Similarly, contemporary neuroscience describes a process called predictive processing, which is about the ability to correct errors in the efface of new information as a way of reorienting actions and thoughts.^49^ The machine learning process might at first appear to be unstable as the repeated running of queries produces different outputs, but in fact, it is mirroring the way that humans process information.

Finally, we have explored the potential application of the software for synthesizing primary study data. One key question is how this kind of synthesis of primary data, using Leximancer or similar approaches, compares with the findings of other forms of evidence synthesis. There is an opportunity for future research to compare empirically the findings of this synthesis of primary study data versus synthesis of primary study findings of the same dataset, such as a meta‐ethnography of associated publications.

### 4.6 Summary

The findings presented here provide an illustration of how Leximancer might help to generate insights, particularly initial, fresh analytical lines of enquiry, from a pooled large qualitative dataset. We have summarized the advantages as the ability to help with “zooming in” and “zooming out” of the data. The disadvantages of using these techniques for pooled primary datasets are largely the considerable time needed to access and prepare data and the need for further interpretative work to provide meaningful outputs. We suggest that machine learning techniques and text analytics software such as Leximancer can facilitate qualitative data synthesis of unusually large datasets in any field but caution that this approach requires more reflection on and critique of its underlying algorithms and assumptions.

## FUNDING

This project is funded by the Academy of Medical Sciences and the Wellcome Trust (Springboard—Health of the Public 2040 [HOP001\1051]). Ruth Garside is partly funded by the National Institute for Health Research (NIHR) Collaboration for Leadership in Applied Health Research and Care (CLAHRC) for the South West Peninsula at Royal Devon and Exeter NHS Foundation Trust. This report is independent research, and the views expressed in this publication are those of the authors and not necessarily those of the NHS, the National Institute for Health Research, or the Department of Health.

## CONFLICT OF INTEREST

The authors declare that they have no competing interests.

## DATA AVAILABILITY STATEMENT

The original transcripts from the primary research studies included in this secondary analysis were only accessible to the authors for the length and use of this project and are therefore not available to third parties; the corresponding author can be of assistance to liaise with the original institutions that hold the data.

## Supporting information

Data S1. Example transcript template

Data S2. Example output ‐ graphs

Data S3a. Example output ‐ theme summary ‘people’

Data S3b. Example output ‐ theme summary ‘people’
