# Supplementary material for: Semiautomated text analytics for qualitative data synthesis
Source: Res Synth Methods. 2019 Jul 9;10(3):452–64. doi: 10.1002/jrsm.1361 (PMC6772124; doi:10.1002/jrsm.1361)
Supplement: Supplementary file 1 — Data S1. Example transcript template [file JRSM-10-452-s001.docx]

**Supporting Information 1: Example transcript template**

**Unique identification number:** Study code; Interview/Focus group; Gender; Number

**Title:** ID. Interview/Focus group. Setting/location. Gender. Age. (ID from primary study)

Include a colon and space after descriptor as below.

**COCIF1 Interview Cambridge Female 30-54 (was CXXXX)**

INTERVIEWER: XXXX

COCIF1: XXXX

INTERVIEWER: XXXX

COCIF1: XXXX

INTERVIEWER: XXXX

COCIF1: XXXX

INTERVIEWER: XXXX

COCIF1: XXXX

INTERVIEWER: XXXX

COCIF1: XXXX

INTERVIEWER: XXXX

COCIF1: XXXX

INTERVIEWER: XXXX

COCIF1: XXXX

INTERVIEWER: XXXX

COCIF1: XXXX
