# Supplementary figures and images for: Semiautomated text analytics for qualitative data synthesis
Source: Res Synth Methods. 2019 Jul 9;10(3):452–64. doi: 10.1002/jrsm.1361 (PMC6772124; doi:10.1002/jrsm.1361)

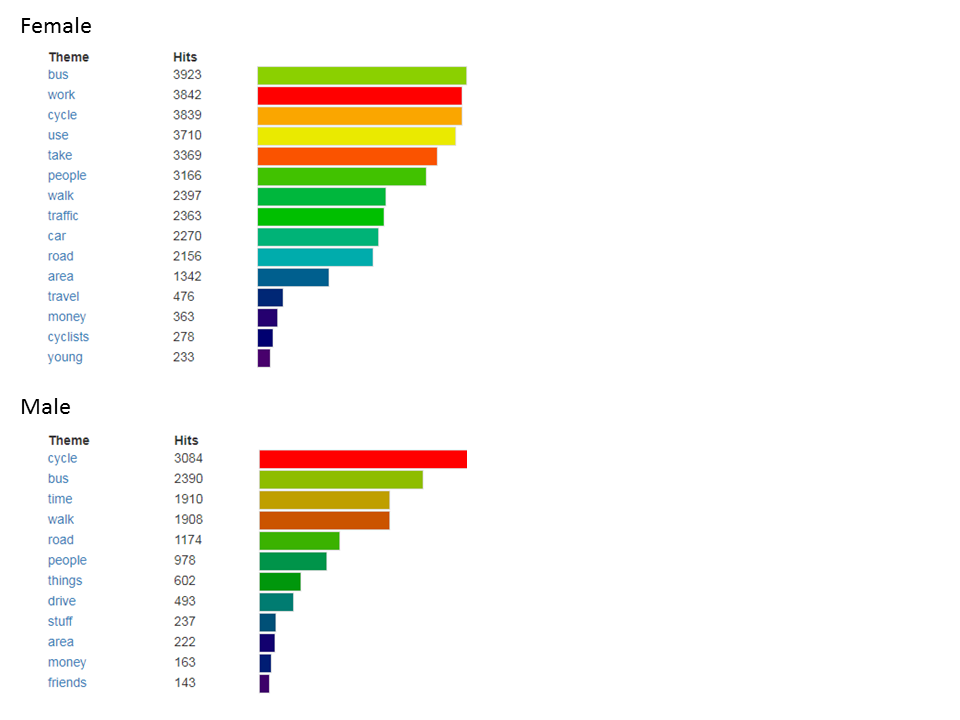

Supplement: Supplementary file 2 — Data S2. Example output ‐ graphs [file JRSM-10-452-s002.tif]

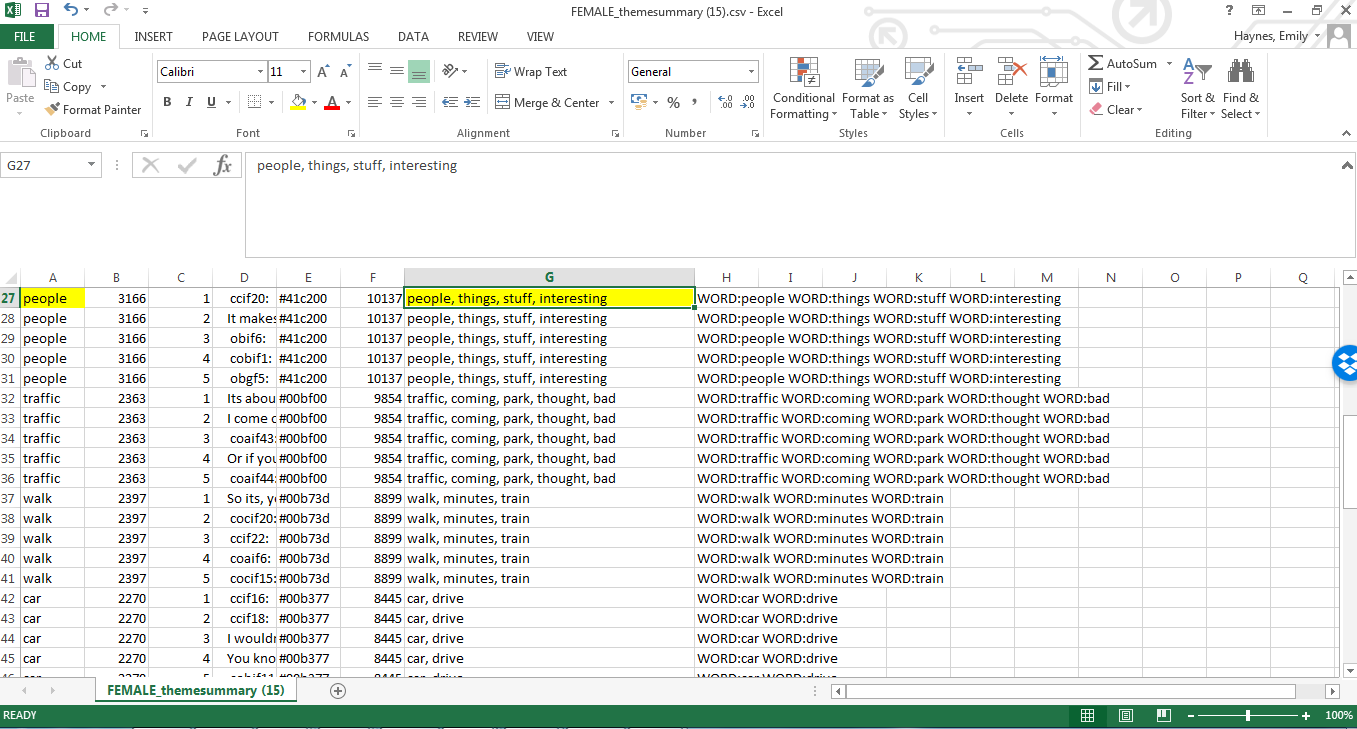

Supplement: Supplementary file 3 — Data S3a. Example output ‐ theme summary ‘people’ [file JRSM-10-452-s003.tif]

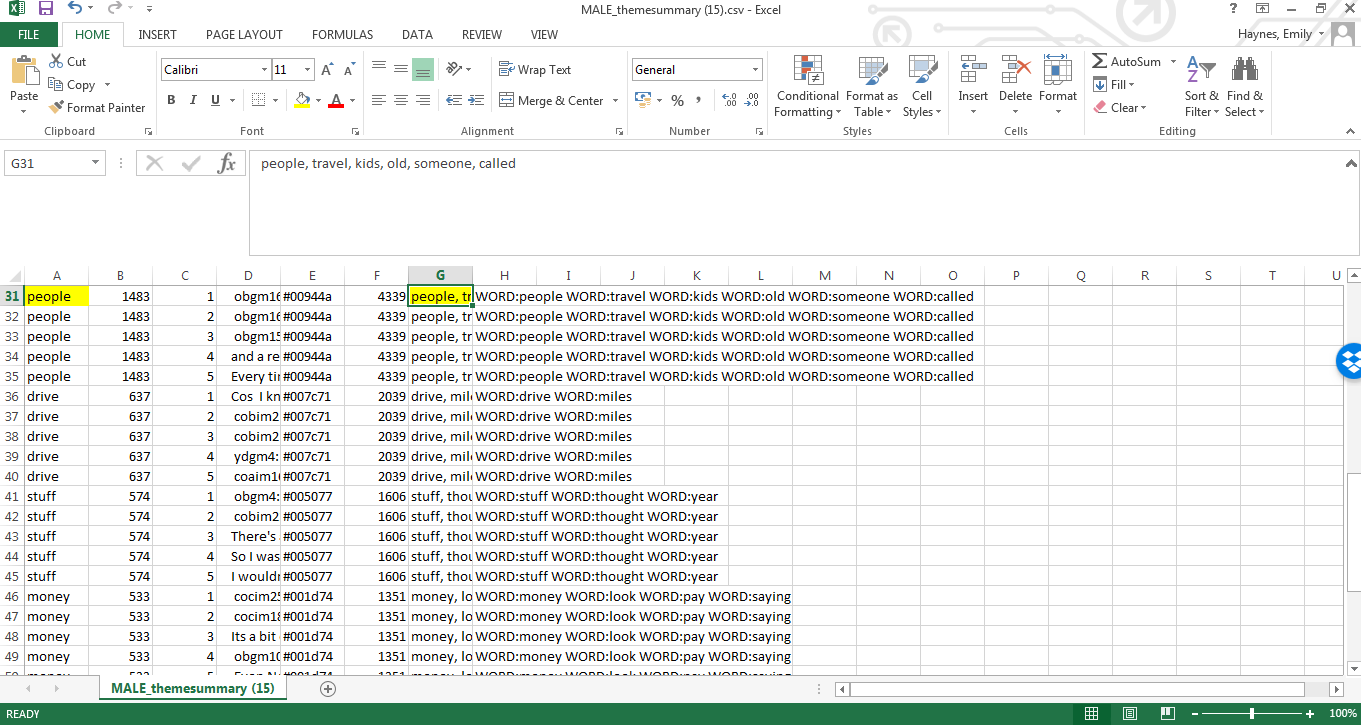

Supplement: Supplementary file 4 — Data S3b. Example output ‐ theme summary ‘people’ [file JRSM-10-452-s004.tif]
